# Lung involvement in children with newly diagnosed rheumatic diseases: characteristics and associations

**DOI:** 10.1186/s12969-022-00731-5

**Published:** 2022-08-20

**Authors:** Hua Huang, Yabin Hu, Yufen Wu, Fei Ding, Xuemei Xu, Yingying Jin, Yanliang Jin, Yixiao Bao

**Affiliations:** 1 Department of Rheumatology and Immunology, Shanghai Children’s Medical Center, School of Medicine, Shanghai Jiao Tong University, No. 1678, Dongfang RD., Pudong district, Shanghai, 200127 China; 2 Department of Clinical Epidemiology and Biostatistics, Shanghai Children’s Medical Center, School of Medicine, Shanghai Jiao Tong University, Shanghai, China; 3 Department of Respiratory Medicine, Shanghai Children’s Medical Center, School of Medicine, Shanghai Jiao Tong University, Shanghai, China; 4 Shanghai Tonxin Clinic, No. 118, Zhengheng RD., Yangpu district, Shanghai, 200000 China

**Keywords:** Lung involvement, Rheumatic disease, Pulmonary function test

## Abstract

**Background:**

Pulmonary complications of rheumatic diseases may cause functional impairment and increase mortality. However, reports regarding detection of lung involvement in children with treatment-naive, newly diagnosed rheumatic diseases are scarce. Herein, we aimed to describe the characteristics of such patients and explore the association between lung involvement and rheumatic disease.

**Methods:**

From January 2019 to June 2021, 48 pediatric patients with treatment-naive, newly diagnosed rheumatic diseases at Department of Rheumatology and Immunology, Shanghai Children's Medical Center, School of Medicine, Shanghai Jiao Tong University were included with pulmonary function tests (PFTs) and high-resolution computed tomography (HRCT) findings, and 51 age-matched healthy controls were examined based on PFTs. Univariate and multivariable logistic regression analyses were used to investigate the clinical characters and laboratory parameters associated with lung involvement in these patients.

**Results:**

Asymptomatic patients had a faster respiratory rate and a higher ratio of forced expiratory volume in 1 s/forced vital capacity than the controls (*P* < 0.05). More patients than controls were observed to have a decreased DL_CO_ below the lower limit of normal (18 of 45 [40.0%] vs. 6 of 36, respectively; *P* = 0.041). Among the 48 patients, 8 (16.7%) had abnormal HRCT findings and 27 (56.3%) had abnormal PFT results. Thirty-one (64.6%) patients had lung involvement. Logistic regression revealed that increases in the erythrocyte sedimentation rate (ESR) and CD4/CD8 ratio were associated with increased odds ratio of lung involvement (1.037, 95% CI: 1.003–1.072; 9.875, 95% CI: 1.296–75.243, respectively).

**Conclusions:**

Pediatric patients with treatment-naive, newly diagnosed rheumatic diseases are prone to pulmonary involvement. Increased ESR and CD4/CD8 are associated with elevated odds of lung involvement in patients. We recommend routine pulmonary evaluation in such patients, especially in high-risk patients, even in the absence of respiratory symptoms, once they are diagnosed with rheumatic disease.

## Background

Rheumatic diseases are a group of immunologically mediated inflammatory disorders affecting multiple organs. Albeit rare, pulmonary diseases can occur in nearly all pediatric rheumatic conditions. Systemic inflammatory diseases with the highest likelihood of pulmonary involvement are juvenile systemic lupus erythematosus (JSLE), scleroderma (systemic sclerosis [SSc]), juvenile dermatomyositis (JDM), mixed connective tissue disease (MCTD), granulomatosis with polyangiitis and juvenile idiopathic arthritis (JIA) [1].

With respect to lung involvement, interstitial lung disease (ILD) is the primary presentation in adult patients with diverse forms of rheumatic disease [2]. Pulmonary artery hypertension (PAH) and obstructive lung disease can also occur [3]. Pulmonary complications are often less frequent in childhood than in adulthood but may also cause functional impairment and increase mortality [4]. The incidence and spectrum of pulmonary manifestations vary among patients with rheumatic conditions. Over 50% of patients with juvenile-onset systemic sclerosis develop ILD [5]. Pleuritis is the most common pulmonary complication, affecting up to 80% of pediatric patients with SLE [6]. However, PAH is uncommon, occurring in up to 14% of patients with JSLE [7]. In patients with JIA, the prevalence of lung disease is estimated to be 4–8% [8]. Although this remains low, children with systemic JIA are being increasingly found to have more severe and life-threatening lung diseases such as PAH, ILD, and pulmonary alveolar proteinosis [9]. Overall, diffuse parenchymal lung disease is most commonly observed in almost every condition in the spectrum of rheumatic diseases. Presentation ranges from mild to acute respiratory failure. Additionally, ILD can be the first manifestation of rheumatic disease. Recognition of pulmonary involvement depends on the methods used to detect the disease. Finding reliable means, especially when patients are asymptomatic, would provide a rationale for early intervention.

However, compared with that on adults, research on detecting pulmonary complications in children with newly diagnosed rheumatic diseases is scarce. Thus, the present study aimed to compare pulmonary involvement in newly diagnosed treatment-naive patients of rheumatic disease with age-matched healthy controls using pulmonary function tests (PFTs) and investigate the characteristics of and associations with lung involvement in such patients.

## Methods

### Patients and controls

This cross-sectional study was conducted between January 2019 and June 2021 at the Department of Rheumatology and Immunology, Shanghai Children's Medical Center, School of Medicine, Shanghai Jiao Tong University. In this study, 48 pediatric patients and 51 aged-matched healthy controls were enrolled. The patients were treatment-naive and had been diagnosed with rheumatic diseases for the first time. Patients with a history or clinical findings of respiratory infection at the time of study or chronic pulmonary diseases (such as asthma) or heart disease were excluded. All patients fulfilled the commonly accepted classification criteria as follows: patients with SLE were classified based on the American College of Rheumatology Classification Criteria for SLE (1982, revised in 1997) [10] and the Systemic Lupus International Collaborating Clinics group classification criteria (2012) [11], patients with JIA fulfilled the International League of Association for Rheumatology Classification of juvenile idiopathic arthritis [12], JDM was diagnosed according to the Bohan and Peter criteria [13], Sjogren’s syndrome (SS) was diagnosed based on the European criteria [14], localized scleroderma (LSS) was diagnosed based on the Pediatric Rheumatology European Society Criteria [15], MCTD was diagnosed based on Kasukawa’s criteria [16], and SSc was diagnosed based on the Pediatric Rheumatology European Society/American College of Rheumatology/European League against Rheumatism provisional classification criteria [17]. The pediatric Behçet's disease classification was used for Behçet's disease [18] and the American College of Rheumatology 1990 criteria was used for the classification of Takayasu arteritis [19]. Undifferentiated connective tissue disease (UCTD) referred to unclassifiable systemic autoimmune diseases which share clinical and serological manifestations with definite CTDs such as SLE, SSc, SS, dermatomyositis/polymyositis, MCTD, and rheumatoid arthritis (RA) but which did not fulfill any existing classification criteria [20].

Age-matched controls were randomly selected from those who underwent health check-ups at the developmental and behavioral clinic of Shanghai Children’s Medical Center. These patients had no medical history of rheumatic, heart, or chronic lung diseases. Physical examination of the lungs revealed no abnormalities.

### Data collection

A data collection format was designed to include the following parameters: age at diagnosis, sex, height, weight, duration between disease onset and diagnosis, history of cigarette exposure, and clinical data including the levels of acute-phase reactants viz. erythrocyte sedimentation rate (ESR) and C-reactive protein (CRP), serological rheumatoid factor (RF), antinuclear antibody (ANA), interleukin-1 (IL-1), IL-6, tumor necrosis factor-a (TNF-a), CD4/CD8 ratio, and vitamin D_3_ (VitD_3_).

Patients and controls were evaluated using PFTs, and pulmonary high-resolution computed tomography (HRCT) was performed only for patients. The maximum vital capacity (VC_max_), forced vital capacity (FVC), forced expiratory volume in 1 s (FEV1), FEV1/FVC ratio, lung diffusion for carbon monoxide (DL_CO_), and forced expiratory flow between 25 and 75% of vital capacity (FEF_25-75_) were recorded. The main respiratory functional parameters were expressed as percentages of the predicted values for age, height, and sex (absolute measured value/predicted value × 100). DL_CO_ was corrected for hemoglobin concentration. A DL_CO_ < 80% of the predicted value was considered abnormal [21]. DL_CO_ was deemed mildly, moderately, or severely reduced if it was 60–80%, 40–60%, or < 40% of the predicted value, respectively [22]. PFTs were measured according to guidelines adopted by the American Thoracic Society and Europe Respiratory Society [23].

Each measurement was repeated at least thrice, and the highest acceptable measurement was compared with the normal predicted values. Pulmonary dysfunction was defined as restrictive dysfunction (VC < 80% of that predicted), obstructive dysfunction (FEV1/FVC < 80% of that predicted and FEV1 < 80% of that predicted), isolated impairment of diffusion capacity (DL_CO_ < 80% of that predicted), or small airway disease (FEF_75_ or FEF_75_, and FEF_50_ < 65% of that predicted).

Two senior experienced radiologists who were blinded to the clinical data read the HRCT images separately. Ground-glass opacity, consolidation, septal thickening, honeycombing, crazy paving, nodules, pleural and pericardial effusions, progressive volume loss, and pulmonary embolism were regarded as the main findings on HRCT [24].

### Statistical analysis

Continuous variables were expressed as mean and standard deviation (SD) or median and interquartile range (IQR; [Q1, Q3]) as appropriate. Categorical variables were presented as the number of cases (percentage). Differences between patients and controls were tested using Student’s t-test for normally distributed values. The Mann–Whitney U-test was used to compare non-normally distributed data between two groups. The chi-square test or Fisher’s exact test was used for categorical variables. Correlations between two variables were determined using Spearman’s coefficient. Univariate and multivariable logistic regression analyses were performed to calculate the odds ratio (OR) and 95% confidence interval (CI) for lung involvement in patients with rheumatic diseases. All statistical analyses were performed using IBM SPSS 26.0 software (IBM, Armonk New York, USA). The statistical significance level was set at *P* < 0.05 (two-sided).

## Results

### Clinical characteristics

The clinical characteristics of the study participants are presented in Table 1. Patients with rheumatic diseases consisted of 29 girls and 19 boys (mean age = 11.3 years, SD = 2.6), and aged-matched healthy controls consisted of 31 girls and 21 boys (mean age = 11.3 years, SD = 1.8). Among these patients, 16 had SLE, 11 had JIA, 5 had JDM, 4 had SS, 4 had LSS, 3 had MCTD, 2 had Behçet’s disease, 1 had UCTD, 1 had Takayasu arteritis, and 1 had SSc. The median duration between disease onset and diagnosis was 0.25 years [0.08, 1]. Two patients had a family history of CTDs: a mother with SLE and a mother with RA. Nineteen patients had passive smoking exposure. Of the 48 treatment-naive patients with newly diagnosed rheumatic disease initially enrolled, 1 was excluded owing to symptoms of chest tightness (she was diagnosed with ILD and SLE later), leaving a cohort of 47 patients with no clinical evidence of pulmonary involvement for comparison with controls.

**Table 1:** Clinical characteristics of the participants

**Variables**	**Patients with rheumatic disease** (*n* = 47)	**Controls** (*n* = 51)	***P-value***
Age (years)	11.3 ± 2.6	11.3 ± 1.8	0.999
Sex (female/male)	28/19	30/21	1
Height (cm)	145.0 [136.0, 153.5]	147.0 [138.3, 158.0]	0.275
Weight (kg)	35.0 [28.4, 47.5]	38.8 [31.9, 49.4]	0.173
Respiratory (times/min)	21.4 ± 2.7	19.6 ± 1.9	0.002*
Heart (beat/min)	90.0 [85.5, 98.0]	90.0 [85.5, 99.0]	0.882

^*^P < 0.05 was considered statistically significant

There were no significant differences in age, height, or weight between patients and controls. Compared with controls, asymptomatic patients had a significantly higher respiratory rate on physical examination (21.4 vs. 19.6, *P* = 0.002), but the absolute values were within normal limits.

### Pulmonary function tests

Results of lung function tests are shown in Table 2. The FEV1/FVC ratio was significantly higher in the group with rheumatic diseases than in the control group (*P* < 0.05). The average VC_max_ (%), FVC (%), FEV_1_ (%), FEF_25-75_ (%), and DL_CO_ (%) were lower than those of the controls. However, this difference was not significant (*P* > 0.05). Eighteen of the 45 patients (40.0%) had a reduction in DL_CO_ below the lower limit of normal (LLN): one (2.2%) with moderate impairment and 17 (37.8%) with mild reduction. Six of the 36 controls (16.7%) had abnormal DL_CO_ values below the LLN. The difference between the two groups was significant (*P* < 0.05; Table 3). There was no significant difference in other indices between the two groups.

**Table 2:** Respiratory parameters

**Variables**	**Patients with rheumatic** **disease** (*n* = 47)	**Controls** (*n* = 51)	***P-value***
VC_max_ (%pred)	95.7 ± 11.5	97.6 ± 9.5	0.371
FVC (%pred)	97.6 [91.8, 106.4]	100.5 [91.7, 107.7]	0.440
FEV_1_ (%pred)	102.5 ± 14.3	104.2 ± 10.1	0.493
FEV_1_/FVC (%pred)	104.0 ± 7.7	98.6 ± 9.7	0.004*
FEF_25_ (%pred)	98.4 ± 17.1	101.3 ± 12.5	0.502
FEF_50_ (%pred)	99.6 ± 24.4	100.1 ± 18.8	0.908
FEF_75_ (%pred)	88.4 ± 29.0	91.1 ± 27.7	0.638
DL_CO_ (%pred)	88.1 ± 17.7	93.7 ± 12.1	0.114

^*^P < 0.05 was considered statistically significant

*Abbreviations*: *VC*_*max*_ Maximum vital capacity, *FVC* Forced vital capacity, *FEV*_*1*_ Flow expiratory volume in 1 s, *FEF*_*25-75*_ Forced expiratory flow at 25% to 75% of the FVC, *DL*_*CO*_ Lung diffusion for carbon monoxide, the values were adjusted for hemoglobin

Note: All the tests are expressed as percentage of theoretical values for sex, age, and height. Continuous variables are presented as mean ± SD. Non-parametric variables were expressed as median and IQR

**Table 3:** Abnormal lung function test findings

	**Number (percentage) of the results below LLN**		
**Parameter**	**Study group (*n* = 47)**	**Control group (*n* = 51)**	***P-value***
VC_max_	5 (10.6%)	3 (5.9%)	0.624
FVC	3 (6.4%)	2 (3.9%)	0.925
FEV_1_	2 (4.3%)	1 (2.0%)	0.943
FEF_25-75_	10 (21.3%)	5 (7.8%)	0.107
DL_CO_	18 (40.0%)	6 (16.7%)	0.041*

^*^P < 0.05 was considered statistically significant

*Abbreviations*: *LLN* Lower limit of normal, *VC*_*max*_ Maximum vital capacity, *FVC* Forced vital capacity, *FEV*_*1*_ Flow expiratory volume in 1 s; FEF_25-75_, forced expiratory flow at 25% to 75% of the FVC, *DL*_*CO*_ Lung diffusion for carbon monoxide

### HRCT findings in patients with rheumatic diseases

HRCT was performed for all patients with rheumatic diseases. Abnormal thoracic HRCT was observed in 8 of the 48 (16.7%) patients, and they were all women (Table 4). There was a significant difference between the sexes (*P* = 0.015). Among these patients, two had SLE, four had JDM, one had SS, and one had JIA. There was only one type of HRCT alteration in six patients. Two types of alterations were simultaneously found on HRCT in two patients. Radiological abnormalities were as follows: ground-glass opacities (*n* = 3), linear opacities (*n* = 1), nodules or micronodules (*n* = 4), septal thickening (*n* = 1), and pleural thickening (*n* = 1). Four of the eight patients (50.0%) also had PFTs abnormalities, including restrictive dysfunction (*n* = 1), small airway disease (*n* = 2), and reduction of DL_CO_ (*n* = 3). HRCT abnormalities were primarily related to rheumatic diseases, and patients with HRCT abnormalities due to infections, drug toxicity, and neoplasia were excluded.

**Table 4:** Characteristics of patients with abnormal HRCT

Age(years)	Sex	Diagnose	PFT abnormal	CT abnormal	Site
12.3	Female	SLE	Severely restrictive dysfunction + small airway disease	Ground-glass opacities	Bilateral
14.3	Female	JDM	Normal	Multiple nodules + ground-glass opacities	Bilateral
12.1	Female	JDM	Mild reduction of DL_CO_	Septal thickening	Left
12.0	Female	JDM	Mild reduction of DL_CO_	Ground-glass opacities	Bilateral
17.2	Female	SLE	Mild reduction of DL_CO_ and small airway disease	Linear opacities	Right
8.0	Female	JDM	Normal	Multiple subpleural nodules	Right
12.6	Female	SS	Normal	Subpleural nodules + pleural thickening	Left
11.8	Female	JIA	Normal	Multiple micronodules	Bilateral

*Abbreviations*: *HRCT* High-resolution computed tomography, *PFT* Pulmonary function test, *CT* Computed tomography, *SLE* Systemic lupus erythematosus, *JDM* Juvenile dermatomyositis, *SS* Sjogren’s Syndrome, *JIA* Juvenile idiopathic arthritis

### Characteristics of patients with lung involvement

Eight of 48 (16.7%) patients with rheumatic diseases had abnormal HRCT, and 27 of 48 (56.3%) patients had abnormal PFTs. In total, 31 patients (64.6%) had lung involvement (Table 5). Among the 31, 22 (71.0%) were girls and 9 of 31 (29.0%) were boys (mean age [SD]: 11.2 [2.5] years). Among these 31 patients, 14 had SLE, 7 had JIA, 4 had JDM, 3 had SS, 2 had LSS, and 1 had Takayasu arteritis. The median duration between disease onset and diagnosis was 0.25 [0.08, 0.625] years. Nine of 31 (29.0%) patients had elevated CRP levels, 19 of 31 (61.3%) patients were positive for ANA, and 3 of 31 (9.7%) patients were positive for RF.

**Table 5:** Differences in rheumatic disease with and without pulmonary involvement

Variables	Pulmonary involvement	Non-pulmonary involvement	*P-value*
Age, mean ± SD (years)	11.2 ± 2.5	11.6 ± 2.7	0.687
Female (%)	22/31 (70.1)	7/17 (41.2)	0.044*
Height (cm)	143.0 ± 12.4	146.8 ± 12.6	0.314
Weight (kg)	36.1 ± 11.1	41.8 ± 12.8	0.111
Illness 1(%)- SLE	14 (45.2)	2 (11.8)	0.025*
Illness 2 (%)- JIA	7 (22.6)	4 (23.5)	
Illness 3 (%)- JDM	4 (12.9)	1 (5.9)	
Illness 4 (%)- others	6 (19.4)	10 (58.8)	
Disease duration, median [Q1, Q3] years	0.25 [0.08, 0.62]	0.58 [0.17, 1]	0.275
Cigarette exposure (%)	44.4	64.3	0.381
ESR, mean ± SD (mm/h)	43.9 ± 31.9	21.5 ± 21.0	0.031*
CRP > 8 mg/L (%)	9/31 (29.0)	2/17 (11.8)	0.316
ANA ( +) (%)	19/31 (61.3)	10/17 (58.8)	0.867
RF ( +) (%)	3/31 (9.7)	2/17 (11.8)	0.789
CD4/CD8, mean ± SD	1.4 ± 0.5	1.0 ± 0.5	0.026*
Medication exposures	NO	NO	

^*^P < 0.05 was considered statistically significant

*Abbreviations*: *SD* Standard deviation, *SLE* Systemic lupus erythematosus, *JIA* Juvenile idiopathic arthritis, *JDM* Juvenile dermatomyositis, *ESR* Erythrocyte sedimentation rate, *CRP* C-reactive protein, *ANA* Antinuclear antibody, *RF* Rheumatoid factor

### Factors associated with lung involvement

Univariate and multivariate logistic regression analyses were performed to identify factors associated with lung involvement (Table 6). Among the 48 patients with rheumatic diseases, 31 (64.6%) had lung involvement (abnormal PFTs and/or abnormal HRCT). Patients were then divided into rheumatic disease groups with or without lung involvement. Sex, age at diagnosis, type of disease, passive smoking, duration from onset to diagnosis, serum CRP, ESR, white blood cell count, hemoglobin level, platelet count, ANA positivity, and RF, IL-1, IL-6, TNF-a, VitD_3_, natural killer cell count, and CD4/CD8 positivity were evaluated for the two groups. Univariate logistic regression revealed that female sex (OR = 3.492, 95% CI 1.012–12.051, *P* = 0.048), elevated ESR (OR = 1.026, 95% CI 1.001–1.052, *P* = 0.039), and increased CD4/CD8 (OR = 5.625, 95% CI 1.145–27.638, *P* = 0.033) were significantly associated with lung involvement. In contrast to other diseases (including SS, LSS, MCTD, Behçet’s disease, UCTD, Takayasu arteritis, and SSc), SLE was associated with increased OR of lung invovlment [11.667, 95% CI 1.940,70.178, *P* = 0.007].

**Table 6:** Factors associated with lung involvement in rheumatic diseases

Variables	Univariate model 1			Multivariate model 1			Multivariate model 2		
	**OR**	**95% CI**	***P-value***	**OR**	**95% CI**	***P-value***	**OR**	**95% CI**	***P-value***
**Age**	0.951	0.751–1.205	0.679	0.812	0.598–1.104	0.183	0.831	0.581–1.188	0.309
**Sex** **(female)**	3.492	1.012–12.051	0.048*	5.702	0.993–32.745	0.051	6.914	0.967–49.420	0.054
**Type of disease**									
**SLE**	11.667	1.940–70.178	0.007*				24.192	2.305–253.960	0.008*
**JIA**	2.917	0.594–14.327	0.187				7.079	0.400–125.228	0.182
**JDM**	6.667	0.597–74.506	0.123				6.106	0.338-110.314	0.220
**Others**	**Ref**						**Ref**		
**ESR**	1.026	1.001–1.052	0.039*	1.037	1.003–1.072	0.032*			
**CD4/CD8**	5.625	1.145–27.638	0.033*	9.875	1.296–75.243	0.027*	6.778	0.770–59.641	0.085

^*^P < 0.05 was considered statistically significant

*Abbreviations*: *OR* Odds ratio, *CI* Confidence interval, *SLE* Systemic lupus erythematosus, *JIA* Juvenile idiopathic arthritis, *JDM* Juvenile dermatomyositis, *CRP* C-reactive protein, *ESR* Erythrocyte sedimentation rate, *WBC* White blood cell, *HB* Hemoglobin, *PLT* Platelet, *ANA* Antinuclear antibodies, *RF* Rheumatoid factor, *IL* Interleukin, *TNF-a* a-tumor necrosis factor, *VitD*_*3*_ Vitamin D_3_, *NK* Natural killer cells

Spearman’s correlation analysis revealed a moderate correlation between ESR and disease type (*P* = 0.002). Therefore, ESR and disease type were separately analyzed in the two multivariable models. In multivariable model 1 (without disease type), elevated ESR (OR = 1.037, 95% CI 1.003–1.072, *P* = 0.032) and increased CD4/CD8 (OR = 9.875, 95% CI 1.296–75.243, *P* = 0.027) remained associated with lung involvement. In multivariable model 2 (without ESR), in contrast to other diseases, SLE were associated with increased OR (24.192, 95% CI 2.305, 253.960, *P* = 0.008) of lung involvement in patients with rheumatic disease.

## Discussion

In this study, we found that lung involvement was quite prevalent among patients with rheumatic disease. Over half of the patients had abnormal PFT and/or abnormal HRCT findings, even though most were asymptomatic. This is similar to the data presented by Peradzyńska et al. [25], who found that 42% of children with newly diagnosed CTDs had asymptomatic pulmonary involvement. These patients had abnormal lung function, which was almost fourfold higher than that of healthy controls.

Many disease-modifying antirheumatic drugs have been reported to cause pulmonary toxicity, such as drug-induced ILD, in many case reports and retrospective studies [26]. Attanasi et al. found a negative correlation of DL_CO_ with cumulative methotrexate (MTX) dosage and the duration of MTX treatment in one study on JIA [8]. All patients in our study were treatment-naive and newly diagnosed. They did not have any history of medication treatment, even though the most extended duration of one patient with LSS was 6 years. Thus, the side effects of the drug on pulmonary diseases could be excluded.

In our study, abnormal lung function was significantly more common than structural changes, as assessed using HRCT. Of the patients, 56.3% had abnormal PFT and 16.7% had abnormal HRCT findings. Fifty percent of patients with abnormal HRCT findings did not have concurrent PFT abnormalities. Lilleby et al. also found that PFT values were often abnormal, and the abnormalities in PFT results were not accompanied by HRCT abnormalities [21]. In contrast to our study, Veiga et al. found that HRCT showed some abnormalities in 70% of patients with childhood-onset SLE, which was minimal 43% [27]. Similar to the results of the structural assessment, the functional evaluation showed a high prevalence of abnormalities in their research. However, many studies on adults have reported an unusually high prevalence of HRCT abnormalities, suggestive of ILD, in asymptomatic SLE patients with normal PFTs [28].

In our study, the most frequent pulmonary dysfunction was a reduction in DL_CO_ values below the LLN, and the FEV1/FVC ratio was significantly higher in patients than in controls.

40% of our cases had a reduction in DL_CO_. Abnormal DL_CO_ has also been described to occur commonly in other research. In a longterm study of patients with JMCD, Hetlevik et al. found low DL_CO_ in 65% of cases [29]. Similarly, A low DL_CO_ in approximately 50% of the JDM patients was found in Sanner H et al.’s research [30]. Novel findings are that JDM-active had lower diffusion capacity than JDM-inactive in Marstein et al.’s study [31].

DL_CO_ is the most sensitive test for predicting the presence of ILD [32], and a reduction in DL_CO_ suggests abnormalities of the alveolar–capillary interface, compatible with diffuse vascular and/or parenchymal lung disease [8]. The significant increase in FEV1/FVC and respiratory rate compared with that of controls may reflect volume loss associated with fibrosis, although FVC and FEV1 remained within the normal range after the decline. Thus, the absence of symptoms and “normal” PFT findings cannot fully rule out mild pulmonary involvement [33].

HRCT abnormalities were found in 16.7% of the patients with airway or parenchymal abnormalities. The frequency of HRCT abnormalities was low in this study. However, as HRCT is the gold standard method for defining specific lung abnormalities [34], Céspedes-Cruz et al. recommended that patients with SSc could undergo both PFTs and HRCT to increase the possibility of early detection of pulmonary disease [35].

When newly diagnosed patients with rheumatic disease at risk of pulmonary involvement need initial evaluation or follow up patients who are unable to tolerate other investigations need to be distinguished from pulmonary diseases, HRCT is recommended. HRCT is a low-dose examination and produces only a 5–10% radiation dose of the conventional contiguous CT [36]. However, as HRCT might be too sensitive as a screening tool for unselected patients, HRCT should be used to confirm or exclude ILD only in high-risk groups or when there is reason to suspect ILD [37].

Based on our cohort of patients with rheumatic diseases, elevated ESR and CD4/CD8 ratio appeared to be significantly associated with pulmonary involvement in the univariate and multivariate logistic regression. These findings suggest some underlying mechanisms of pulmonary disease development in patients with rheumatic diseases. Inflammatory proteins in the lungs are associated with progressive lung disease [38]. In a prospective cohort of patients with RA, Paulin et al. found a good correlation between ESR and ground-glass score on HRCT [39]. This reflects the inflammatory nature of ground-glass opacities. This association between systemic inflammation and lung disease suggests that better control of the disease process lowers the risk of progressive lung involvement.

Turesson et al. reported a significantly increased number of CD4 + cells compared with those in patients with interstitial pneumonitis (IP) in a study of lung biopsy tissue from RA-associated idiopathic IP [40]. However, there was a less striking difference in the CD8 + subset, suggesting that CD4 + T cells are critical for developing pulmonary manifestations in RA. Similar findings were reported in adult patients with SS [41]. In the present study, we confirmed that CD4 + T cells are a correlated factor for lung involvement in children with rheumatic disease.

Our study did not find an association between disease duration and lung involvement. This is different from the study by Dai et al., where showed that patients with a longer duration of symptoms before SLE diagnosis tended to have pulmonary participation [42]. Alkady et al. revealed that there was a significant inverse correlation between disease duration and lung function parameters [43]. However, they explained that long-term MTX treatment and a history of severe disease activity might be associated with abnormalities in lung function. Therefore, disease activity and medication use may correlate with lung involvement instead of disease duration.

SLE, JIA, and JDM were the most common diseases observed in our cohort. In logistic regression analysis of factors associated with lung involvement, diseases with few cases were regardes as one group. We found SLE was significantly associated with increased odds of lung involvement compared to other diseases. Owing to the considerable heterogeneity and a small number of cases, further studies are needed to confirm this.

In our study, all patients with abnormal HRCT were female. Based on univariate logistic regression, female sex was associated with lung involvement in rheumatic disease. It is well known that, in the adult population, CTDs are far more common in women. Although this sex dimorphism is less common in children, JIA, JDM, SLE, SSc, and Takayasu arteritis are more common in girls than in boys [44]. Whether estrogen or other factors are involved in the mechanism of lung involvement in patients with rheumatic diseases requires further investigation.

This study has some limitations. First, the sample size of the study was relatively small, since it was challenging to recruit pediatric patients with treatment-naive, newly diagnosed rheumatic diseases. Second, the study groups showed significant heterogeneity. The different clinical entities were unevenly distributed. Third, our hospital is one of three children’s hospitals in Shanghai. Owing to location and other reasons, there may be a selection bias of illness among different hospitals. Therefore, multi-center longitudinal studies are necessary to identify lung involvement in different rheumatic diseases, response to treatment, and prognosis in the future and our results should be verified preferably in the follow-up studies. Nonetheless, to the best of our knowledge, this is the largest study on pulmonary involvement in Chinese children with treatment-naive, newly diagnosed rheumatic diseases and the first exploration of the factors associated with lung involvement in pediatric patients.

## Conclusion

Lung involvement is relatively common in children with treatment-naive, newly diagnosed rheumatic diseases. Using PFTs and HRCT, we identified patients with asymptomatic lung involvement. Patients at higher risk may be identified using ESR and CD4/CD8 ratios. Regular pulmonary appraisal as part of the routine assessment may help monitor these patients and optimize treatments and lung management from the initial stages to avert progression to advanced stages.

## Data Availability

All data generated or analysed during this study are included in this published article.

## Abbreviations

CRP: C-reactive protein
DL_CO_: Lung diffusion for carbon monoxide
ESR: Erythrocyte sedimentation rate
FEF_25-75_: Forced expiratory flow between 25 and 75% of forced vital capacity
FEV1: Forced expiratory volume in 1 s
FVC: Forced vital capacity
IL: Interleukin
ILD: Interstitial lung disease
IP: Interstitial pneumonitis
JDM: Juvenile dermatomyositis
JIA: Juvenile idiopathic arthritis
JSLE: Juvenile systemic lupus erythematosus
LSS: Localized scleroderma
MCTD: Mixed connective tissue disease
MTX: Methotrexate
PAH: Pulmonary artery hypertension
PFT: Pulmonary function test
RA: Rheumatoid arthritis
RF: Rheumatoid factor
SLE: Systemic lupus erythematosus
SS: Sjogren’s syndrome
SSc: Scleroderma (systemic sclerosis)
TNF-a: Tumor necrosis factor-a
UCTD: Undifferentiated connective tissue disease
VC_max_: Maximal vital capacity
